# Putative Factors That May Modulate the Effect of Exercise on Liver Fat: Insights from Animal Studies

**DOI:** 10.1155/2012/827417

**Published:** 2011-09-08

**Authors:** Faidon Magkos

**Affiliations:** ^1^Department of Nutrition and Dietetics, Harokopio University, 17671 Athens, Greece; ^2^Division of Endocrinology, Diabetes, and Metabolism, Beth Israel Deaconess Medical Center, Harvard Medical School, Boston, MA 02215, USA; ^3^Department of Geriatrics & Nutritional Science, Center for Human Nutrition, Washington University School of Medicine, Campus Box 8031, Saint Louis, MO 63110, USA

## Abstract

An increase in intrahepatic triglyceride (IHTG) content is the hallmark of nonalcoholic fatty liver disease (NAFLD) and is strongly associated with insulin resistance and dyslipidemia. Although regular aerobic exercise improves metabolic function, its role in regulating fat accumulation in the liver is incompletely understood, and human data are scarce. Results from exercise training studies in animals highlight a number of potential factors that could possibly mediate the effect of exercise on liver fat, but none of them has been formally tested in man. The effect of exercise on IHTG content strongly depends on the background diet, so that exercise is more effective in reducing IHTG under conditions that favor liver fat accretion (e.g., when animals are fed high-fat diets). Concurrent loss of body weight or visceral fat does not appear to mediate the effect of exercise on IHTG, whereas sex (males versus females), prandial status (fasted versus fed), and duration of training, as well as the time elapsed from the last bout of exercise could all be affecting the observed exercise-induced changes in IHTG content. The potential importance of these factors remains obscure, thus providing a wide array of opportunities for future research on the effects of exercise (and diet) on liver fat accumulation.

## 1. Introduction

Excessive accumulation of fat in the liver, that is, intrahepatic triglyceride (IHTG), is associated with increased prevalence rates of and risk for dyslipidemia, diabetes, and cardiovascular disease [1–3]. Data from epidemiological as well as metabolic studies indicate that increased IHTG content is accompanied by insulin resistance and dysregulation of lipid metabolism [4–6]. Exercise is known to improve metabolic function [7, 8]; however its effects on IHTG remain elusive [9, 10]. Data from studies in humans are scarce and not entirely consistent [11]. In this paper, the results from a number of animal studies are briefly reviewed in an attempt to highlight putative factors that may modulate the effect of exercise on IHTG content.

## 2. Exercise Training in Animals

**Figure 1:**
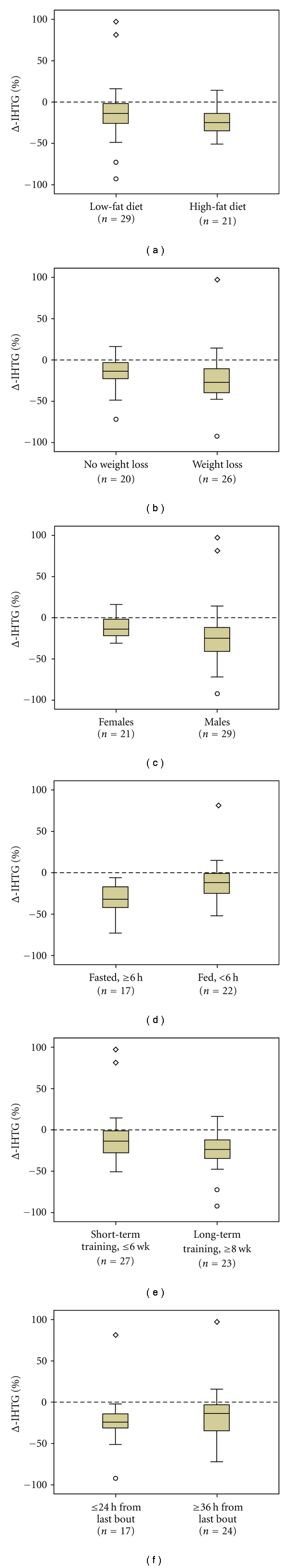
Factors that may affect changes in liver fat in response to exercise training in animals. Exercise-induced changes in intrahepatic triglyceride content (Δ-IHTG) are shown for: (a) animals fed high fat or standard chow (low fat) diets; (b) animals that experienced weight loss (or attenuated weight gain) or not; (c) male or female animals; (d) fasted or fed animals; (e) animals trained for longer or shorter periods of time; (f) animals examined within one day from the last bout of exercise or later during recovery. Box plots have been constructed using average changes in liver fat (% difference relative to sedentary controls) for each group of animals in the studies depicted in Table 1, and illustrate median, first, and third quartiles, minimum and maximum values, as well as potential positive and negative outliers.

Many studies have evaluated the effect of aerobic exercise training on IHTG content in rodents; their design varies in terms of sex, strain, background diet, training duration, the prandial status, and the time of assessment after the last bout of exercise (Table 1) [12–37]. Results are largely heterogeneous, but a crude analysis of the data suggests that endurance training decreases IHTG (median: −16%, range: −92% to +97%, *n* = 50 studies; Table 1). Most frequently [14, 16, 18, 20, 26, 31, 35] but not always [15, 17, 34, 37], exercise has been shown to be more effective in reducing liver fat or attenuating its accretion in animals fed high-fat rather than standard, low-fat diets (median decrease: 25% and 14%, resp., Figure 1(a)). This is consistent with data from human studies, in which exercise training appears to be more potent in reducing IHTG in subjects with increased baseline IHTG, for example, subjects with NAFLD, type II diabetes, or the elderly [11].

The reasons why exercise is more effective in reducing IHTG on high-fat than low-fat diets are not entirely clear but are likely related to the hepatosteatotic effect of high-fat feeding. Fat is mainly stored as microvesicles (<1 *μ*m^2^) within hepatocytes, whereas the high-fat diet-induced hepatic steatosis occurs via accumulation of macrovesicles (>1 *μ*m^2^) [18, 38]. Endurance training has been shown to completely prevent the high-fat diet-induced hepatic steatosis [18, 38], that is, the hepatocyte surface area occupied by the lipid vacuoles, solely by reducing the number of lipid vacuoles in all sizes between 1 and 10 *μ*m^2^ (i.e., macrovesicles), without affecting the number of vacuoles of surface area <1 *μ*m^2^ (i.e., microvesicles) [18]. Hence exercise may have less of an effect when on low-fat diets, not only because of lower total IHTG content, but also because most of this fat (*∼*75%) is stored in microvesicles, not macrovesicles. A more pronounced IHTG-depleting effect of exercise has also been observed under other conditions that favor the development of fatty liver, such as overfeeding [34], ovariectomy [28], ethanol ingestion [39], or tumor-bearing [24]. Apart from the fat content of the background diet, the type of dietary carbohydrate [12], protein [25], and fat (i.e., saturated or unsaturated fatty acids) [22], as well as the feeding pattern (ad libitum or paired) [12] do not appear to affect, at least not in a major way, the exercise-induced change in IHTG content. 

The collective of available data in animals highlights a number of other putative factors that may modulate the effect of exercise on liver fat; however, none of these factors has been formally tested using rigorous experimental designs. Concurrent weight loss or attenuated weight gain is not likely critical for the exercise-induced depletion of IHTG to manifest, albeit they may lead to greater reductions in liver fat when compared to no weight loss or similar weight gain (median decrease: 27.5% and 14%, resp., Figure 1(b)). However, just like in humans [11], loss of visceral adipose tissue mass with exercise training is not necessarily coupled with a corresponding decrease in liver fat [16, 18, 28, 37]. Likewise, human studies have shown that exercise-induced reductions in IHTG content can occur in the absence of changes in total body fat [40] or even visceral adipose tissue [41]. 

Exercise may be more effective in reducing IHTG content in males than in females (median decrease: 25% and 14%, resp., Figure 1(c)), in fasted (≥6 h) than in fed animals (median decrease: 31% and 11%, resp., Figure 1(d)), and after longer (≥8 wk) than shorter interventions [34] (median decrease: 24% and 14%, resp.; Figure 1(e)). The time elapsed from the last bout of exercise (≤24 h or ≥36 h) may also mediate the observed changes in IHTG (median decrease: 24% and 13.5%, resp., Figure 1(f)), suggesting that even acute exercise could affect liver fat. However, relevant information is scarce and inconclusive. A single bout of aerobic exercise (30–60 min) did not affect IHTG content, measured immediately after exercise, in female rats [42] but caused a *∼*30% decrease in male rats [43] of the same strain, under both standard and high-fat feeding conditions. This is in line with data from exercise training studies in animals raising the possibility that males may be more sensitive to the IHTG-reducing effect of exercise than females (Figure 1(c)), as well as with recent observations in humans [44]. Studies in which male rats were exercised until exhaustion provide conflicting results, some observed a mild [45] or marked [46, 47] increase in hepatic steatosis whereas others found a decrease of 30–60% [48] at the end of exercise.

## 3. Detraining after Regular Exercise

If regular exercise reduces liver fat, cessation of exercise should lead to an increase in IHTG content. Only a few animal studies have evaluated the effect of detraining on liver fat accumulation, and all have demonstrated that cessation of regular exercise (after 6–16 weeks of training) for a short (2-3 days) or a long (6 weeks) period of time is not associated with any significant changes in IHTG content compared with the trained state (i.e., before discontinuation of exercise) when animals are fed a standard low-fat diet [28, 36, 49, 50]. Furthermore, detraining for 2–7 days does not alter the total number of hepatocyte lipid vacuoles and their size, even though it does activate precursors and processes in the liver known to initiate steatosis (e.g., decreased mitochondrial oxidative capacity, increased expression of de novo lipogenesis proteins, and increased malonyl CoA levels) [49]. Interpretation of detraining data is not straightforward, though. It is possible that this lack of an effect of detraining relates to the lesser potency of exercise in reducing liver fat content in animals fed standard low-fat diets (Figure 1), so that changes after detraining are similarly less pronounced. For instance, two [28, 36] out of three studies that reported no effect of detraining on liver fat also failed to observe a training-induced decrease in IHTG content, suggesting that training and detraining have no effect on liver fat accumulation under low-fat feeding conditions. Whereas one study [49] did observe a training-induced decrease in liver fat in rats fed a low-fat diet but found no changes after detraining, implying that the IHTG-depleting effect of regular exercise is long-lived and is not readily reversed by detraining. Still, compared with sedentary, never-exercised counterparts, detrained animals appear to be relatively protected from mild hepatic steatosis induced by 2 weeks of high-fat feeding [36], but not from the development of frank fatty liver 6 weeks after ovariectomy [28], even though cessation of exercise training in ovariectomized rats resulted in a nearly 40% increase in IHTG content compared with ovariectomized rats who did not stop exercising [28]. It is thus possible that the relevant molecular and biochemical adaptations to exercise are readily reversed when the exercise routine is interrupted (<1 week), however, changes in IHTG manifest later in time and only when strong triggering factors for liver fat accretion coexist, such as high-fat feeding or ovariectomy. Support for this notion is provided by an earlier study, where detraining resulted in a striking increase in IHTG content only when animals were subjected to starvation and refeeding [50].

## 4. Conclusion

The effect of exercise on IHTG content has recently attracted much scientific interest in light of the apparent detrimental metabolic effects of excessive liver fat accumulation. Although the results from a few studies in human subjects are promising, as exercise appears to reduce IHTG [11], the importance of the factors highlighted herein on the basis of studies in animals has never been evaluated in man. Future studies should at least control for—in order to avoid confounding—or directly investigate the role of these factors in affecting the exercise-induced changes in liver fat content.

## Figures and Tables

**Table 1:** Effect of aerobic exercise training on liver fat in animals.

					Withdrawal before		Effect of training (EX versus	
Study	Animals	Intervention			measurements		respective SED group on the same diet)	
		Diet	Exercise	Duration	Food	Exercise	BW/VAT	Liver fat
Ahrens et al., 1972 [12]	Male Wistar rats; young and mature	HF (ad libitum or pair-fed) with two different carbohydrate sources	SED or EX (1/d, running, 30 min at *∼*13 m/min and 0% incline)	8 wk	12 h	?	↓/? (both diets, feeding patterns, and age groups)	Young: −15% Mature: −35%

Barakat et al., 1987 [13]	Female rats; control and alloxan-diabetic	SC? (ad libitum)	SED or EX (1/d, running, 2 h at 20 m/min and 0% incline)	7 d	0 h	24 h	*↔*/? (both groups)	Control: −14% (*NS*) Diabetic: −2% (*NS*)

Cha et al., 1999 [14]	Male Sprague-Dawley rats	SC or HF (ad libitum)	SED or EX (1/d, running, 1.5 h at 150 m/min and 1% incline)	1 mo	?	?	SC: ↓/? HF: ↓/?	SC: −24% HF: −40%

Chapados et al., 2009 [15]	Female Sprague-Dawley rats	SC or HF (ad libitum)	SED or EX (5/wk, running, progressive until 1 h at 26 m/min and 10% incline for the last 4 wk)	8 wk	12 h	48 h	SC: *↔*/↓ HF: *↔*/↓	SC: −16% (*NS*) HF: −22% (*NS*)

Charbonneau et al., 2005 [16]	Female Sprague-Dawley rats	SC or HF (ad libitum)	SED or EX (6/wk, running, progressive until 1 h at 26 m/min and 10% incline for the last 3 wk)	6 wk (+2 wk diet lead-in)	2-3 h	48 h	SC: *↔*/↓ HF: *↔*/↓	SC: 0% (*NS*) HF: −14%

Fukuda et al., 1991 [17]	Male Wistar rats	SC, HF or HChol (ad libitum)	SED or EX (voluntary running)	4 wk	3 h	?	SC: ↓/? HF: ↓/? HChol: ↓/?	SC: −1% (*NS*) HF: +14% (*NS*) HChol: −30% (*NS*)

Gauthier et al., 2003 [18]	Female Sprague-Dawley rats	SC or HF (ad libitum)	SED or EX (5/wk, running, progressive until 1 h at 26 m/min and 10% incline for the last 4 wk)	8 wk	2 h	48 h	SC: *↔*/↓ HF: ↓/↓↓	SC: +16% (*NS*) HF: −29%

Gauthier et al., 2004 [19]	Female Sprague-Dawley rats	HF (ad libitum)	SED or EX (5/wk, running, progressive until 1 h at 26 m/min and 10% incline for the last 4 wk)	8 wk (+8 wk diet lead-in)	2 h	48 h	*↔*/↓	−16% (*NS*)

Gollisch et al., 2009 [20]	Female Sprague-Dawley rats	SC or HF (ad libitum)	SED or EX (voluntary running)	4 wk	10 h	24 h	SC: *↔*/↓ HF: ↓/↓↓	SC: −16% (*NS*) HF: −31%
Hao et al., 2010 [21]	Female Sprague-Dawley rats; sham-operated or OVX with and without E2	SC (ad libitum)	SED or EX (5/wk, running, 1 h at 18 m/min and 0% incline)	12 wk	?	24 h	Sham: *↔*/*↔* OVX: ↓/↓ OVX+E2: *↔*/*↔*	Sham: −24% OVX: −30% OVX+E2: −5% (*NS*)

Karanth and Jeevaratnam, 2009 [22]	Male Wistar rats	HF rich in SFA or MUFA (ad libitum) with carnitine or not	SED or EX (6/wk, swimming, 1 h)	6 mo	Overnight	20 h	*↔*/? (both diets)	SFA: −35% MUFA: −25% (average with or without carnitine)

Lessard et al., 2007 [23]	Male Sprague-Dawley rats	HF (ad libitum)	SED or EX (5/wk, running, progressive until 1 h at 32 m/min and 15% incline for the last 3 wk)	4 wk (+4 wk diet lead-in)	8–12 h	36–48 h	↓/↓	−41%

Lira et al., 2008 [24]	Male Wistar rats; control and tumor-bearing	SC (ad libitum)	SED or EX (5/wk, running, progressive until 1 h at 20 m/min and 0% incline for the last 2 wk)	8 wk	?	24 h	↓/? (both groups)	Control: −30% Tumor: −92%

Morifuji et al., 2006 [25]	Male Sprague-Dawley rats	SC with casein or soya as protein source (ad libitum)	SED or EX (6/wk, swimming, 2 h)	2 wk	Nonfasting	24 h	↓/↓ (both groups)	Casein: −21% Soya: −24%

Narayan et al., 1975 [26]	Male Holtzman rats	SC or HF (ad libitum)	SED or EX (5/wk, running, progressive until 80–85 min at 23 m/min and 8.5% incline for the last 3 wk)	6 wk	Nonfasting	24 h	?/?	SC: +81% (*NS*) HF: −51%

Petridou et al., 2005 [27]	Male Wistar rats	SC (ad libitum)	SED or EX (voluntary running)	8 wk	6 h	12 h	?/?	−12% (*NS*)

Pighon et al., 2010 [28]	Female Sprague-Dawley rats	SC (ad libitum)	SED or EX (5/wk, running, progressive until 1 h min at 26 m/min and 10% incline for the last 4 wk)	6 wk	3 h	48 h	*↔*/↓	+1% (*NS*)

Pighon et al., 2010 [28]	Female Sprague-Dawley rats; sham-operated or OVX with and without E2	SC (ad libitum)	SED or EX (5/wk, running, progressive until 1 h min at 26 m/min and 10% incline for the last 3 wk)	5 wk	3 h	48 h	↓/↓ (all groups)	Sham: −1% (*NS*) OVX: −26% OVX+E2: −5% (*NS*)
Rector et al., 2008 [29]	Male OLETF rats (obese and diabetic)	SC (ad libitum)	SED or EX (voluntary running)	16 wk	5 h	48 h	↓/↓	−45%
Rothfeld et al., 1977 [30]	Male Sprague-Dawley rats	HF (pair-fed)	SED or EX (voluntary running)	3 wk	?	?	?/?	−14%

Straczkowski et al., 2001 [31]	Male Wistar rats	SC for wk 0–3 and SC or HF for wk 4–6 (pair-fed)	SED or EX (6/wk, running, 3 h at 20 m/min and 10% incline)	6 wk	?	48 h	↓/? (both diets)	SC: +97% HF: −9% (*NS*)

Terao et al., 1987 [32]	Male Wistar rats	HChol (ad libitum)	SED or EX (5/wk, running, progressive until 1 h at 20 m/min and 0% incline for the last 2 wk)	5 wk (+5 wk SC diet lead-in)	?	?	↓/?	−16% (*NS*)

Tsutsumi et al., 2001 [33]	Male Sprague-Dawley old rats	SC (ad libitum)	SED or EX (1/d, running, 30 min at 15 m/min and 10% incline)	3 mo	*∼*12 h	*∼*12 h	↓/↓	−41%

Vieira et al., 2009 [34]	Male C57BL/6, HF diet-induced obese mice	SC or HF (ad libitum)	SED or EX (5/wk, running, 40 min at 12 m/min and 12% incline)	6 wk	12 h	48 h	SC: *↔*/*↔* HF: ↓/↓	SC: −49% (*NS*) HF: −11% (*NS*)

Vieira et al., 2009 [34]	Male C57BL/6, HF diet-induced obese mice	SC or HF (ad libitum)	SED or EX (5/wk, running, 40 min at 12 m/min and 12% incline)	12 wk	12 h	48 h	SC: *↔*/↓ HF: ↓/↓	SC: −72% HF: −48%

Vieira et al., 2009 [35]	Male Balb/cByJ mice (with defective fatty acid oxidation)	SC or HF (ad libitum)	SED or EX (5/wk, running, 1 h at 12 m/min and 5% incline)	12 wk	12 h	48 h	SC: *↔*/? HF: ↓/?	SC: −5% (*NS*) HF: −40% (*P* = 0.09)

Yasari et al., 2006 [36]	Female Sprague-Dawley rats	SC (ad libitum)	SED or EX (5/wk, running, progressive until 1 h at 26 m/min and 10% incline for the last 4 wk)	8 wk	3 h	48 h	*↔*/↓	−9% (*NS*)

Yasari et al., 2010 [37]	Female Sprague-Dawley rats	SC for wk 0–6 and SC or HF for wk 7-8 (ad libitum)	SED or EX (5/wk, running, progressive until 1 h at 26 m/min and 10% incline for the last 4 wk)	8 wk	3 h	36–48 h	*↔*/↓ (both diets)	SC: −13% (*NS*) HF: +7% (*NS*)

All changes shown are statistically significant versus control group (SED), unless indicated otherwise (*↔* is unchanged; ↓ is reduced; *NS* is not significant; ? is unknown).

BW: body weight; E2: estradiol; EX: exercised; HChol: high fat and cholesterol; HF: high fat; MUFA: monounsaturated fatty acids; OVX: ovariectomized; SC: standard chow (low fat); SED: sedentary; SFA: saturated fatty acids; VAT: visceral adipose tissue (mesenteric, retroperitoneal, and/or epididymal fat pads).
